# Molecular detection and isolation of Lumpy Skin Disease Virus from outbreak cases in Ilubabor Zone, Oromia, Ethiopia

**DOI:** 10.1186/s12866-026-05112-6

**Published:** 2026-05-11

**Authors:** Shimelis Befekadu Abdissa, Abde Aliy Mohammed, Tesfaye Rufael Chibssa, Dereje Shegu Gebrewold, Isayas Asefa Kebede, Morka Dandecha Bayu

**Affiliations:** 1 School of Veterinary Medicine, Ambo University, P.O. Box 19, Guder, Ethiopia; 2 Researcher in the Molecular and Genomics Laboratory in the Animal Health Institute, Sebata, Ethiopia; 3 General Director of Animal Health Institute, Sebata, Ethiopia; 4 Researcher in Virology in the Animal Health Institute, Sebata, Ethiopia; 5 College of Veterinary Medicine and Agriculture, Addis Ababa University, Bishoftu, Ethiopia

**Keywords:** Lumpy skin disease virus, Virus isolation, Real-time polymerase chain reaction, Darimu, Bacho, Yayo, Ilubabor

## Abstract

**Background:**

Lumpy Skin Disease (LSD) is a viral disease caused by Lumpy Skin Disease Virus (LSDV), a member of the genus *Capripoxvirus* within the family *Poxviridae*. It is a notifiable, economically important disease of cattle that causes significant production losses in affected areas.

**Methods:**

A descriptive outbreak investigation was conducted from October 2021 to August 2022 in Darimu, Bacho, and Yayo districts of Ilubabor Zone, Western Oromia, Ethiopia. The study aimed to detect and isolate LSDV from clinically affected cattle. A total of 79 samples were collected from 22 clinically affected cattle, including skin nodule biopsies (*n = *17), skin scrapings (*n = *7), whole blood (*n = *17), and swabs (ocular *n = *14, nasal *n = *12, and lesion swabs *n = *12). Viral DNA was extracted using the QIAGEN kit and detected by real-time polymerase chain reaction (qPCR). Virus isolation was attempted in lamb kidney cell culture from 17 skin nodule biopsies.

**Results:**

Out of 1,080 cattle at risk, 146 were affected, and 7 died. Overall, morbidity, mortality, and case fatality rates were 13.5%, 0.65%, and 4.8%, respectively. All 79 samples (100%) tested positive by real-time qPCR**.** Seventeen skin nodule biopsies were selected for virus isolation and inoculated into lamb kidney cell culture. Ct values varied by sample type: skin nodules (mean Ct 19.96), lesion swabs (21.95), and skin scrapes (21.31) had the lowest Ct values (indicating higher viral load), while blood had the highest (31.69). Cytopathic effects were observed in 4 of the 17 (23.5%) cultures. The changes included cell rounding, aggregation of degenerating cells, and monolayer detachment.

**Conclusion:**

Lumpy Skin Disease Virus was confirmed as the cause of the investigated outbreak, resulting in economic losses for farmers in the study area. Regular annual vaccination and further studies on field viruses and vaccine strain compatibility (matching) are recommended to support disease control and improve eradication strategies.

**Supplementary Information:**

The online version contains supplementary material available at 10.1186/s12866-026-05112-6.

##  Introduction

Ethiopia hosts Africa’s largest livestock population, with about 65 million cattle, 91 million sheep and goats, 8 million camels, and 49 million chickens [1]. The livestock sector contributes up to 40% of agricultural Gross Domestic Product (GDP), nearly 20% of total GDP, and 20% of national foreign exchange earnings [2]. However, its full economic potential is constrained by the high burden of infectious and transboundary animal diseases [3, 4]. These diseases reduce production and productivity through morbidity, mortality, and trade and movement restrictions**,** thereby affecting rural livelihoods and national economic performance [5].

LSD is a major transboundary viral disease of cattle in sub-Saharan Africa, including Ethiopia, with frequent outbreaks reported despite vaccination efforts. This notifiable disease is considered economically devastating by the World Organisation for Animal Health. It causes severe production losses through reduced milk yield, infertility, abortion, mastitis, skin damage, and occasionally death. The causative agent is LSDV, a member of the genus *Capripoxvirus* within the family *Poxviridae* [6].

LSD was first reported in Zambia in 1929 and subsequently spread to Botswana in 1943 and other African countries [7, 8]. It was later reported outside Africa in several Middle Eastern countries in the late 1980 s [9]. In Ethiopia, LSD was first documented in 1981 in the northwestern region near Lake Tana [10] and has since been reported in multiple parts of the country [11]. Frequent outbreaks are associated with seasonal peaks in arthropod vector activity under warm and wet conditions, and are facilitated by cattle movement, communal grazing, and pastoral production systems [12]. Reported outbreaks include 1,352 cases between 2007 and 2011 [13] and 1,015 between 2012 and 2015 [14], totaling 2,367 outbreaks nationwide.

Clinically, LSD is characterized by fever, generalized skin nodules, lesions in internal organs such as the mouth and lungs, keratitis, conjunctivitis, lymphadenopathy, and edema of the limbs and brisket [15]. Transmission occurs mainly through mechanical transmission by arthropod vectors, including mosquitoes and hard tick species [16].

Diagnosis of LSD is based on virus isolation, electron microscopy, serological assays such as ELISA and virus neutralization tests, and molecular methods including PCR [17–19]. However, serological methods do not reliably differentiate *Parapoxvirus* and *Capripoxvirus* species**,** limiting their diagnostic value [20]. Real-time PCR provides rapid, sensitive, and specific detection of *Capripoxvirus* DNA and is widely used for outbreak confirmation [21]. Although melting curve analysis and high-resolution melting PCR can aid differentiation, definitive distinction between LSDV and other *Capripoxviruses* (e.g., goatpox and sheeppox) often requires molecular characterization or sequencing [22, 23].

In endemic countries such as Ethiopia, LSD control relies mainly on vaccination. Several live attenuated *Capripoxvirus* vaccines are used, including LSDV Neethling strain, Kenyan sheep and goat pox virus (KSGPV) strains O-240 and O-180, Yugoslavian RM65, and Romanian and Gorgan strains [24]. Despite these efforts, recurrent outbreaks and suspected vaccine failures continue to be reported [22, 25], suggesting that the epidemiology of LSD in Ethiopia is not fully understood.

Although numerous outbreaks have been reported across Ethiopia, most confirmations are based on clinical diagnosis alone. Laboratory-confirmed data from several high-risk areas, including Ilubabor Zone, remain limited or unavailable**.** This zone is characterized by high cattle density, a warm and humid climate favorable for arthropod vectors, and active cattle movement along trade routes, all of which facilitate LSD transmission. However, no systematic study combining molecular detection and virus isolation has been conducted in this area.

Clinical diagnosis alone is unreliable, as LSD can be confused with other diseases presenting similar skin lesions [26]. Although PCR has been applied in previous studies, very few investigations in Ethiopia have combined real-time PCR with virus isolation to confirm active infection in outbreak settings in the current study area**.** Such combined approaches are important to distinguish field infections from vaccine-related reactions and to better understand circulating strains. Furthermore, robust laboratory-based surveillance is essential to guide vaccination strategies and movement control measures. Without such evidence from high-risk zones like Ilubabor, control efforts remain largely reactive rather than evidence-based.

Therefore, this study aimed to detect and isolate LSDV from outbreak cases in Ilubabor Zone using real-time PCR and cell culture, to generate laboratory-based evidence of active field infection.

## Materials and methods

### Study areas

**Fig. 1:**
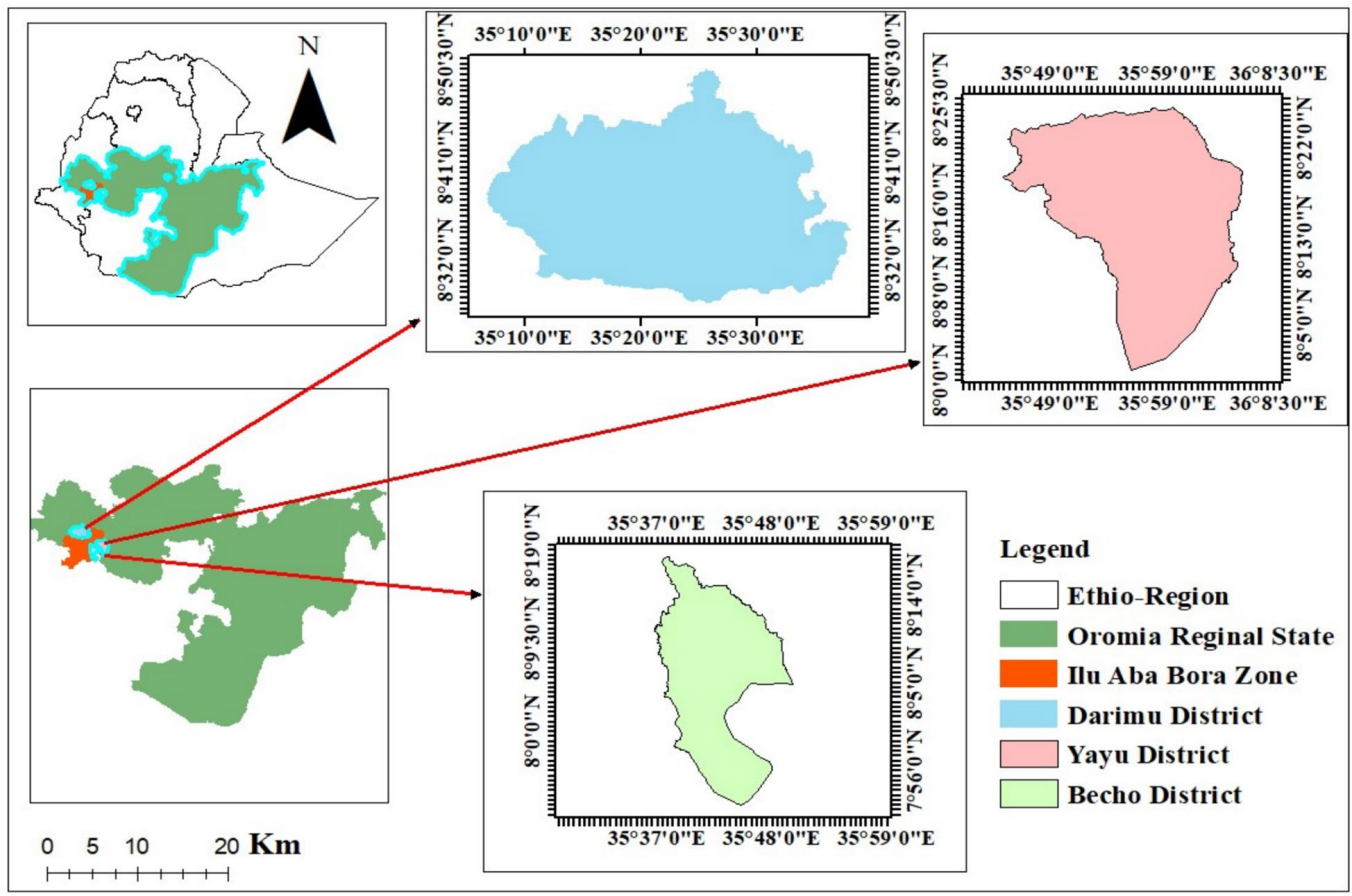
Map showing study areas (ArcGIS 10.8, 2026)

The study was carried out in Darimu, Bacho, and Yayo districts of Ilubabor Zone, Southwestern Oromia, Ethiopia (Fig. 1). These districts were selected based on reported LSD outbreaks from district and peasant association (PA) animal health experts. The study focused on three districts and five PAs. Ilubabor Zone is located in southwestern Ethiopia, approximately 600 km from Addis Ababa, the capital city. The zone comprises 14 districts and covers an area of about 9,278.82 km^2^, lying between 8.27526°N latitude and 35.75596°E longitude. The elevation ranges from 500 to 2,575 m above sea level. Agroecologically, the zone is classified as Dega (10%), Woina Dega (67%), and Kolla (23%). The mean annual temperature ranges from 18 °C to 24 °C, while annual rainfall varies between 1,150 and 2,200 mm. The livestock population of the zone is estimated to include 1,966,280 cattle, 539,840 sheep, 207,752 goats, 68,420 horses, 15,040 mules, 62,850 donkeys, and 1,895,450 poultry (Ilubabor Zone Agricultural [27]).

### Study population

The study population consisted of local and crossbred cattle of both sexes and all age groups in Ilubabor Zone, Oromia Region, Ethiopia. The animals were managed under a traditional extensive production system by smallholder farmers. Cattle were purposively selected from herds reporting suspected LSD outbreaks during the study period. The sampled animals were privately owned, and verbal consent was obtained from the owners before clinical examination and sample collection. Only cattle meeting the clinical case definition for LSD were included. No healthy control animals were included; therefore, this study does not allow estimation of subclinical infection or population-level prevalence**.**

### Study design

A descriptive outbreak investigation study was conducted from October 2021 to August 2022 to detect and isolate LSDV from clinically suspected cases.

### Sample size determination and sampling techniques

Data on active outbreak cases, including the number of infected animals, deaths, and total animals at risk, were collected from district and peasant association (PA) veterinary clinic experts.

#### Case definition

A suspected LSD case was defined using a standardized clinical case definition based on published guidelines [28, 29]. An animal was considered a suspected case if it exhibited at least two of the following clinical signs: firm, circumscribed skin nodules on the neck, back, perineum, or limbs; fever; enlargement of superficial lymph nodes; ocular or nasal discharge; and/or keratitis, conjunctivitis, or limb/brisket edema. Only animals meeting this case definition were eligible for sampling.

#### Sample size and sampling

A total of 22 cattle were included, comprising 11 from Darimu, 9 from Bacho, and 2 from Yayo districts. Sample size was not statistically calculated**,** but was determined by the number of clinically suspected cases available during the outbreak investigation period, reflecting field constraints during active outbreaks. A total of 79 samples were collected from the 22 suspected cattle for molecular detection and virus isolation, following OIE guidelines [30]. Multiple sample types were collected per animal to improve diagnostic sensitivity, in line with OIE recommendations [29]. These included: 17 skin nodule biopsies, 17 whole blood samples, 7 skin scrapings, and 38 swabs (14 ocular, 12 nasal, and 12 from ruptured skin nodules).

A purposive sampling approach was used. Only cattle that met the clinical case definition were selected. This targeted sampling was designed to maximize the likelihood of detecting LSDV in a confirmatory study. The sampling strategy deliberately oversampled animals with strong clinical suspicion of LSD and excluded asymptomatic or mildly affected animals. Consequently, the sample is not representative of the general cattle population in the study area, and the observed proportion of PCR-positive animals (100%) cannot be interpreted as an estimate of true prevalence or incidence. Instead, the high positivity rate reflects the efficacy of the clinical case definition in identifying true LSD cases and underscores the utility of combining field diagnosis with laboratory confirmation. The primary value of this sampling approach lies in generating a set of well-characterized, laboratory-confirmed cases from which LSDV isolates could be obtained for further molecular characterization, an essential step for surveillance and vaccine matching, not for prevalence estimation. Therefore, the findings, including PCR positivity rates, should not be interpreted as prevalence estimates for the study area.

### Data collection

#### Clinical examination

Data were collected during active outbreak investigations from each animal meeting the clinical case definition. A structured data collection form was used to record demographic variables, including age, sex, breed, and vaccination history. A detailed physical examination was performed on each animal before sample collection. Clinical signs suggestive of LSD, including skin nodules, lymph node enlargement, lameness, and fever, were recorded during field examination. All data were recorded on paper forms at the time of examination and later entered into Microsoft Excel for data management.

#### Specimen collection and transportation

Samples for molecular detection and virus isolation were collected within 1–4 weeks after the onset of clinical signs during outbreak investigations. Suspected cattle were handled gently with the assistance of owners, and a purposive sampling technique was employed.

All samples were collected from unvaccinated animals where vaccination history was confirmed through owner reporting and local veterinary records. Samples were not uniformly collected from each animal. No skin nodule biopsies or skin scrapings were collected from the Yayo district due to lack of owner consent. Additionally, ocular and nasal swabs were collected only from animals showing relevant clinical signs. Nodular lesions were sampled only when they were ruptured and contained exudate.

To improve diagnostic yield, at least three preferred sample types were collected from each suspected animal from different anatomical sites, in line with recommendations by Tuppurainen et al. [29]. Samples were collected aseptically in viral transport medium (VTM) containing phosphate-buffered saline, antibiotics, and antifungals, using sterile Falcon tubes. Whole blood (5 mL per animal) was collected aseptically from the jugular vein into EDTA vacutainer tubes.

Each sample was labeled with a unique identification number and immediately placed in an icebox with ice packs for transport. Samples were transported to the Animal Health Institute (AHI), Sebeta, Ethiopia. Upon arrival, samples were stored at − 20 °C, while whole blood samples were kept at 4 °C until processing.

### Laboratory diagnosis

#### DNA extraction

Lumpy skin disease virus DNA was extracted from homogenized skin nodule biopsies, skin scrapes, whole blood, and ocular, nasal, and lesion swabs using a Qiamp DNA Mini Kit (Qiagen, Germany) according to the manufacturer’s instructions at the Animal Health Institute molecular biology laboratory. For skin scrapes and nodule biopsies, samples were ground using sterile mortars and pestles with the addition of sterile sand (baked at 200 °C for 4 h to inactivate potential contaminants) to facilitate mechanical disruption. Swab samples were squeezed to release their contents, and whole blood was equilibrated to room temperature. All samples were centrifuged at 2,000 rpm for 2 min, and 200 μL of the supernatant was transferred to a new tube. Next, 20 μL of proteinase K and 200 μL of AL lysis buffer were added, mixed by vortexing, incubated at 56 °C for 10 min, and centrifuged. Then, 200 μL of absolute ethanol (96–100%) was added, and the mixture was mixed. The mixture was applied to a QIAamp Mini spin column and centrifuged at 8,000 rpm for 1 min. The column was washed with 500 μL of AW1 buffer (centrifuged at 8,000 rpm for 1 min) and 500 μL of AW2 buffer (centrifuged at 14,000 rpm for 3 min). DNA was eluted with 200 μL of AE buffer, incubated for 1 min, and centrifuged at 8,000 rpm for 1 min; the elution was repeated. The extracted DNA was stored at −20 °C until amplification. To confirm DNA quality, the extracted samples were analyzed by agarose gel electrophoresis before PCR.

#### Real-time polymerase chain reaction (qPCR)

Rapid laboratory confirmation of LSD was achieved using real-time PCR (qPCR) specific for capripoxviruses, combined with clinical history [30]. Real-time PCR (qPCR) assays targeted the Capripoxvirus genome using specific primers previously validated for the detection of LSDV in Ethiopian field samples [22, 23]. PCR reactions were performed in a 20 μL volume containing: 2 μL forward primer (CP HRM1sb forward: 5' GGT GTA GTA CGT ATA AGA TTA TCG TAT AGA 3’), 2 μL reverse primer (CP HRM1 reverse: 5’ AAT TTC TTT CTC TGT TCC ATT TG 3’), 4 μL nuclease-free water, 10 μL EvaGreen Supermix (BioRad), and 2 μL DNA template. Negative (nuclease-free water) and positive (LSDV standard) controls were included in separate wells. Each sample was tested in duplicate technical replicates to ensure reproducibility, and a mean cycle threshold (Ct) value was calculated for each sample. PCR was performed on a BioSystem 7500 machine with an initial denaturation at 95 °C for 3 min, followed by 45 cycles of denaturation at 95 °C for 15 s, and annealing/extension at 58 °C for 80 s. Melting curve analysis was conducted at 95 °C for 15 s, 40 °C for 1 min, and 85 °C for 15 s [22]. Samples were considered positive if they produced amplification curves with Ct values < 40 and a melting temperature consistent with the positive control (73 °C). The Ct cutoff of 40 was adopted based on the original assay validation [22]. This threshold has been widely used for capripoxvirus real-time PCR to balance diagnostic sensitivity and specificity [31, 32]. Samples with Ct values ≥ 40 were considered negative.

##### Quality control

To prevent contamination, DNA extraction was performed in a dedicated pre-PCR room, physically separated from the PCR setup and post-amplification areas. For each extraction batch, a blank extraction control (nuclease-free water processed through the entire extraction procedure) was included. In each PCR run, two no-template controls (nuclease-free water added to the master mix instead of DNA) and one positive control were included. A run was considered valid only if the positive control produced a Ct value within the expected range and all negative controls showed no amplification. No amplification was observed in any extraction blank or no-template control throughout the study, confirming the absence of contamination.

#### Virus isolation

Lumpy skin disease virus isolation was carried out using a continuous lamb kidney cell line (LK. They were originally obtained from the National Veterinary Institute**,** Ethiopia, a well-known producer of veterinary vaccines and biological products in Africa.LK cells are considered the cell culture system of choice for capripoxviruses due to their high susceptibility and established use in routine virus isolation from field samples [16, 30]. Cells were cultured in Dulbecco’s Modified Eagle’s Medium (DMEM) supplemented with antibiotics and 5% fetal calf serum in 25 cm^2^ tissue culture flasks. Confluent monolayers were detached using trypsin, transferred into sterile 24-well plates (17 wells for viral inoculation, 1 well as a negative control), and incubated at 37 °C for 24 h to allow cell growth before virus inoculation. For each sample, 100 μL of processed homogenate or whole blood was inoculated onto the cell monolayer. Plates were incubated at 37 °C with 5% CO₂ and examined daily for cytopathic effect (CPE), characterized by rounding, detachment, and cell lysis, typically appearing 3–7 days post-inoculation. Cultures showing CPE were harvested, and isolates were passaged up to three times to amplify virus stocks while minimizing the risk of phenotypic alterations associated with extensive in vitro passage [30]. If no CPE was observed after 10 days, a second blind passage was performed,samples failing to produce CPE after two blind passages were considered negative for viable virus. Because CPE alone is not definitive for LSDV, all isolates showing characteristic CPE were subjected to confirmation using LSDV-specific real-time PCR on harvested culture supernatants. Only samples positive by PCR were recorded as confirmed LSDV isolates.

#### Sample preparation and inoculation

Skin nodules, scabs, and crusts contain relatively high amounts of LSDV [28]. In this study, 17 skin nodule biopsy samples that tested positive by PCR were selected for virus isolation on LK cells. Cold chain integrity was maintained throughout transport and storage; samples were transported in an icebox with ice packs. The time from collection to processing ranged from 24 to 72 h, with the majority processed within 48 h, consistent with OIE guidelines for maintaining viral viability [30]. For processing, samples were thawed at room temperature, washed three times with sterile PBS (pH 7.2) containing antibiotics and antifungals to minimize bacterial and fungal contamination. Samples were then cut into pieces using sterile forceps and scissors and homogenized using a sterile mortar and pestle in a Class II biosafety cabinet to prepare 10% (w/v) suspensions.

The suspension was filtered through a 0.45 μm membrane filter (Millipore, USA) and centrifuged at 600 × g for 15 min [33]. Then, 0.5 mL of each filtered supernatant was inoculated onto confluent LK monolayers (17 wells), with one well left uninoculated as a negative control. Plates were incubated at 37 °C for 1 h to allow virus adsorption. Subsequently, 9 mL of Glasgow Minimum Essential Medium (GMEM) containing 0.1% gentamicin and 2% fetal calf serum was added to each well. Inoculated plates were incubated at 37 °C in a humidified incubator with 5% CO₂. Cells were monitored daily using an inverted microscope to observe cytopathic effects (CPE), which is indicative of viral growth [28, 34].

### Data management and analysis

Data from both field and laboratory investigations were screened, entered, and managed using Microsoft Excel. Data were summarized using descriptive statistics, and all statistical analyses were performed using STATA version 12. Morbidity rate was calculated as the number of diseased animals divided by the total number of animals at risk, multiplied by 100. Mortality rate was calculated as the number of deaths divided by the total number of animals at risk, multiplied by 100. The case fatality rate was calculated as the number of deaths divided by the number of clinically affected animals, multiplied by 100. The chi-square (χ^2^) test was used to assess the association between categorical variables (sex, breed, and age) and disease status. A *P*-value of < 0.05 was considered statistically significant.

## Results

### Outbreak investigation and clinical signs

**Fig. 2:**
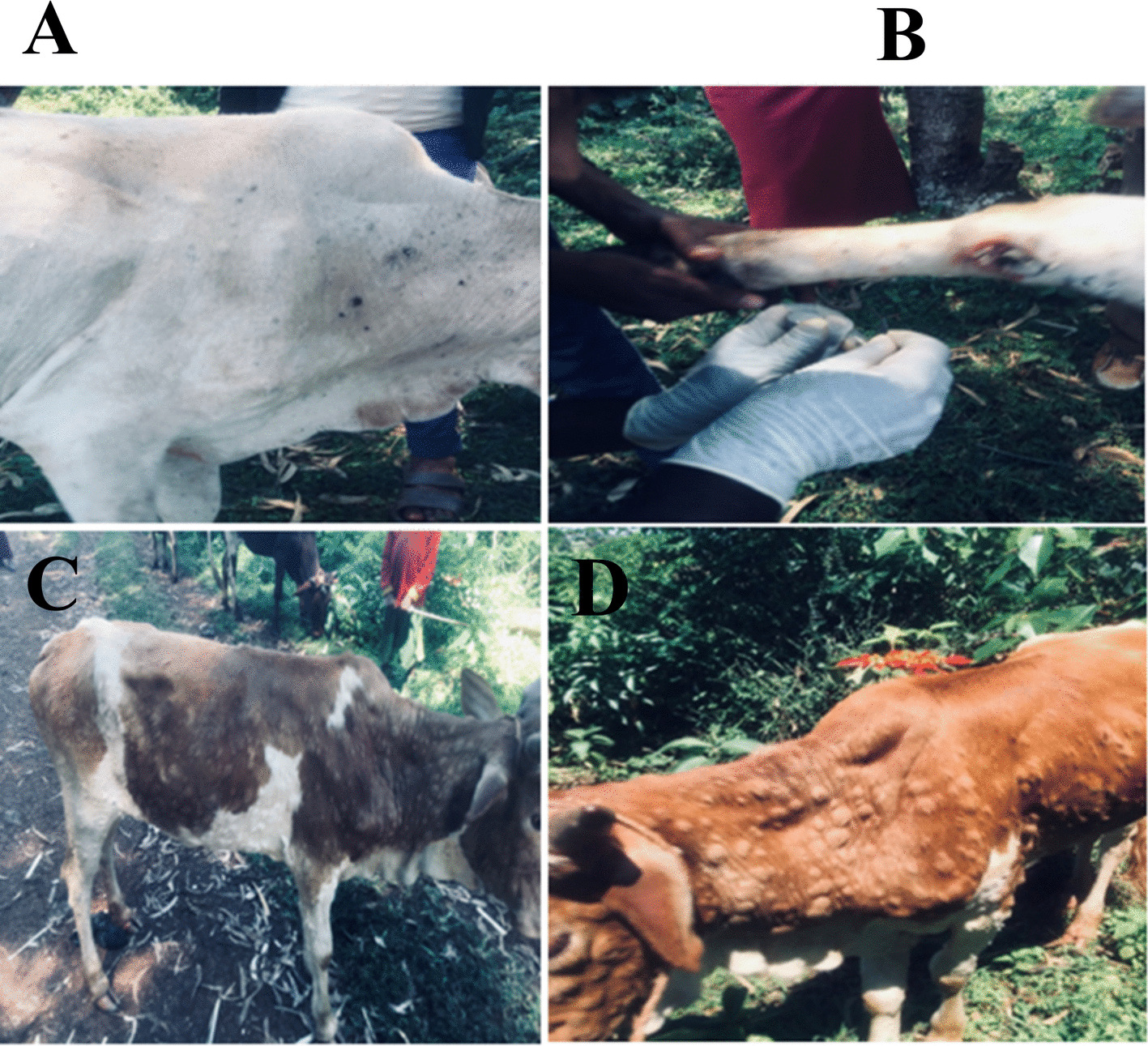
Clinical signs of LSD in affected cattle. **A** Scab formation on the skin; **B** Nodular lesion with crust and pus; **C**, **D** Multiple nodules of varying sizes on the head, neck, and legs

During the outbreak, the most commonly observed clinical signs included multiple skin nodules of varying sizes on the head, neck, and limbs; nodular lesions with purulent discharge and deep scab formation; lacrimation; nasal discharge; lameness; and enlargement of superficial lymph nodes (Fig. 2).

#### Overall morbidity, mortality, and case fatality rates

A total of 1,080 cattle were clinically examined during the outbreak investigations. The overall morbidity, mortality, and case fatality rates were 13.5%, 0.65%, and 4.8%, respectively (Table 1).

**Table 1:** Summary of morbidity, mortality, and case fatality rates in the study districts and peasant associations (PAs)

Districts	PA	Cattle at risk	Affected catle	Morbidity rate (%)	Number of deaths	Mortality rate (%)	Case fatality rate (%)
Darimu	Sharo	230	58	25.2	5	2.17	8.62
	Jarso	175	37	21.1	0	0.00	0.00
	Total	**405**	**95**	**23.5**	**5**	**1.2**	**5.3**
Bacho	Bacho	300	23	7.66	2	0.66	6.7
	Atar	220	15	6.8	0	0.00	0.00
	Total	**520**	**38**	**7.3**	**2**	**0.4**	**5.3**
Yayo	Iluaba-Dinka	**155**	**8**	**5.2**	**0**	**0.00**	**0.00**
Total		**1080**	**146**	**13.5**	**7**	**0.65**	**4.8**

Morbidity, mortality, and case fatality rates were significantly higher in females (18.7%) than in males (9.68%) (Table 2).

**Table 2:** Morbidity, mortality, and case fatality rates by age, sex, and breed of cattle

Factors	Number of cattle at risk	Number of affected	Morbidity rate (%)	Number of deaths	Mortality rate (%)	χ^2^	*P*-Value	Case fatality rate (%)
Age								
1–2 years	146	13	9	0	0.00			0.00
> 2 years	934	133	14	7	0.75	3.08	0.079	5.3
Sex group								
Male	620	60	9.68	2	0.32			3.33
Female	460	86	18.7	5	1.2	18.37	0.000	5.8
Breed								
Local	939	134	14.27	6	0.64			4.5
Crossbreed	141	12	8.51	1	0.7	3.48	0.062	8.33
Total	**1080**	**146**	**13.5**	**7**	**0.65**			**4.8**

### Laboratory confirmation

#### Real-time PCR results

**Fig. 3:**
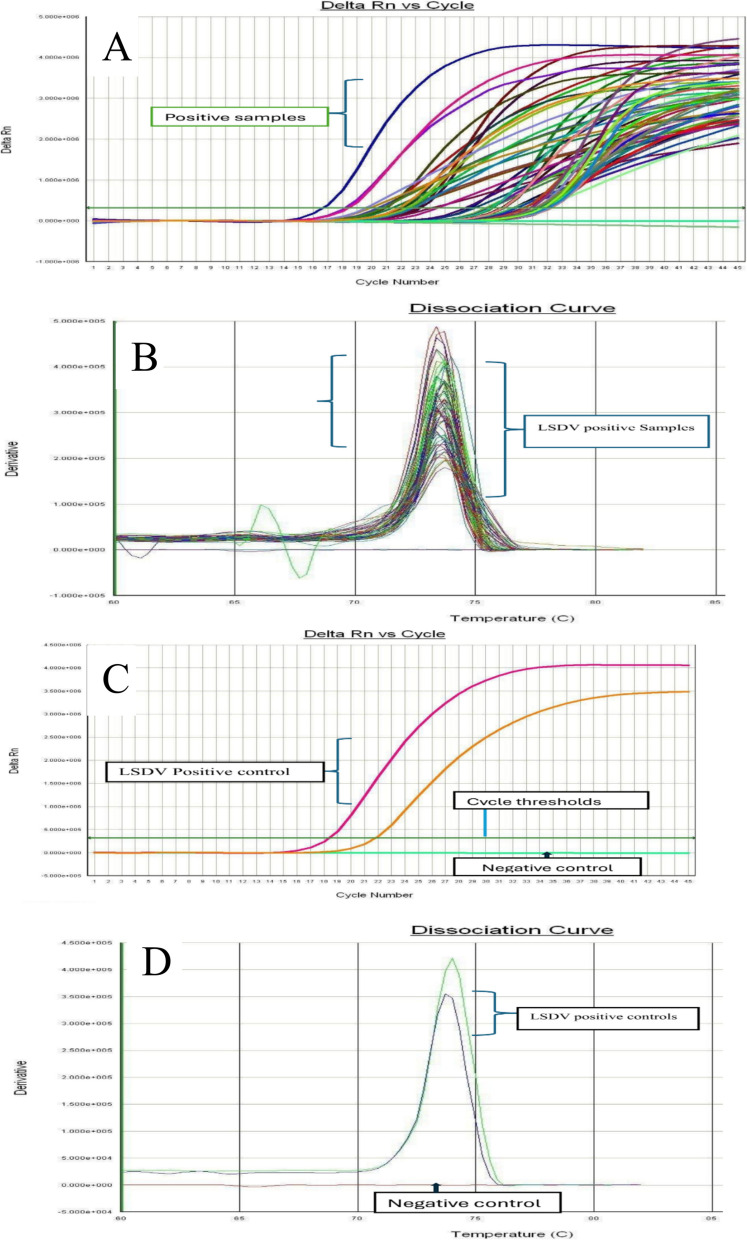
Real-time PCR results of LSDV-positive samples.** A**, **C** Amplification curves; **B**, **D** Melting (dissociation) curves

DNA extracted from 79 clinical samples was tested by real-time PCR, and all samples (100%) were positive for LSDV, with Ct values ranging from 16.29 to 32.27. The LSDV positive control had a Ct value of 18.36, while the negative control (nuclease-free water) showed no amplification. Melting curve analysis confirmed positive samples with temperatures between 73 °C and 74 °C, consistent with the positive control (Fig. 3; Supplementary Material 1).

Analysis of Ct values by sample type showed marked differences in viral load (Table 3). Skin nodules had the lowest mean Ct value (19.66 ± 2.60), indicating higher viral DNA concentration, whereas blood samples had the highest mean Ct value (31.58 ± 0.55), indicating lower viral detection levels.

**Table 3:** Summary of Ct values by sample type

Sample Type	**N**	**Mean Ct ± SD**	**Minimum – Maximum**
Skin Nodule	17	19.96 ± 2.60	16.53–25.50
Skin Scrap	7	21.31 ± 3.41	16.29–26.72
Lesion Swab	12	21.95 ± 3.50	16.69–31.41
Ocular Swab	14	26.43 ± 3.39	20.96–30.82
Nasal Swab	12	27.55 ± 3.16	21.41–31.37
Blood	17	31.69 ± 0.47	30.89–32.53

#### Virus isolation

**Fig. 4:**
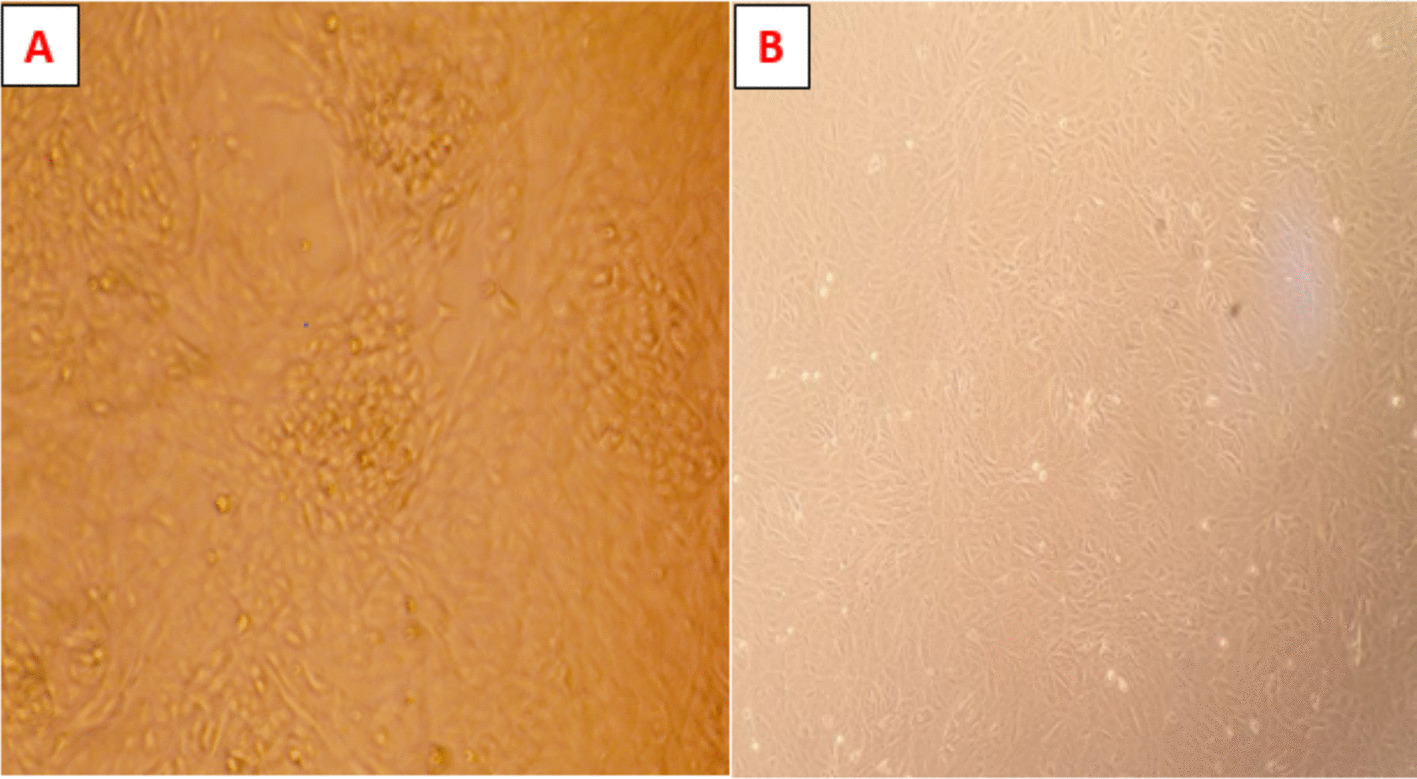
Virus isolation of LSDV in lamb kidney cells. **A** Infected cells showing rounding, aggregation, and detachment of the monolayer; **B** Negative control showing no cytopathic effect

Seventeen skin nodule biopsy samples that tested positive by real-time PCR (qPCR) were selected for virus isolation using lamb kidney (LK) cell culture. Of these, 23.5% (4/17) showed cytopathic effects (CPE) characteristic of LSDV. CPE was first observed during the second passage in all four positive cultures, appearing at day 5 post-inoculation. In the third passage, CPE became more pronounced within 72 h, characterized by cell rounding, aggregation of degenerated cells, and detachment of the monolayer (Fig. 4A). No cytopathic effects were observed in the negative control wells after two blind passages (Fig. 4B). The four isolates were obtained from samples with lower Ct values.

## Discussion

LSD is a serious skin disease of cattle. The present study combined clinical examination, real-time PCR, and virus isolation to investigate LSD outbreaks in the Ilubabor zone from 3 districts of the zone. LSD is an economically important transboundary viral disease of cattle characterized by cutaneous and systemic manifestations. The clinical signs observed were multiple nodules of varying sizes on the head, neck, and legs; nodular lesions with discharging pus and deep scab formation; lacrimation; nasal discharge; lameness; and enlargement of superficial lymph nodes (Fig. 2). These clinical manifestations reflect the dermotropic nature of the LSD virus and its preferential replication in skin and lymphoid tissues. These findings are consistent with previous reports [34–38]. Necrotic nodules, crusted nodules with pus, deep scab formation, and lameness were also observed, aligning with symptoms documented by Leliso et al. [39] and OIE [30]. The observed lesions in this study indicate that the skin serves as the primary target organ for LSDV replication, explaining the prominence of nodular lesions during infection.

The morbidity, mortality, and case fatality rates were 13.5%, 0.65%, and 4.8%, respectively. These values indicate a moderate outbreak impact, characterized by widespread morbidity but relatively low mortality in the affected cattle population. These rates are comparable to the 13.5% morbidity reported by Brenner et al. [8] but lower than rates reported by Leliso et al. [39] (18% morbidity, 1.34% mortality, 7.44% case fatality) and Guyassa et al. [38] (15.49% morbidity, 1.4% mortality, 9% case fatality). Higher morbidity (up to 100%) has been reported in highly susceptible, unvaccinated herds in Egypt [40], underscoring that outbreak severity can vary substantially depending on geography, climate, management practices, immune status, breed, and viral strain [7]. Such variation reflects the multifactorial nature of LSD epidemiology, where host immunity, vector abundance, and management systems play key roles in determining disease severity.

District-level variation was observed, with Darimu showing the highest morbidity (23.5%) and mortality (1.2%), while Bacho had the case fatality rate of 5.3%, compared to Yayo (0.0%). These differences likely reflect variations in geography, climate, and local management practices. In addition, such heterogeneity may be influenced by differences in vector density, cattle movement patterns, and access to veterinary interventions between districts, indicating localized epidemiological dynamics within the outbreak area.

Our study showed that adults are more affected (14%) than young cattle (9%). This aligns with Kasem et al. [41], who reported a 3.4% morbidity in adults, and Guyassa et al. [38], who found higher morbidity in adults (17.18%) compared to the young. The higher morbidity in adults reflects greater lifetime exposure to vectors. This increased exposure risk is likely due to prolonged contact with biting arthropod vectors over time, which serve as mechanical transmitters of LSDV. Conversely, other studies have reported higher morbidity in young cattle [34, 39], suggesting that age-related susceptibility may vary with local conditions and exposure patterns. Age-specific analysis in this study also revealed that mortality and case fatality rates were higher in adults (0.75% and 5.3%, respectively) than in young cattle (0%), likely because adults experience more frequent exposure to risk factors and disease amplification. Lower susceptibility of calves to biting flies may also contribute to the reduced incidence in young animals [42]. Additionally, differences in immune system maturity and prior exposure history may influence age-related disease expression. Nonetheless, young animals may occasionally show severe clinical disease depending on natural susceptibility.

The morbidity rate was higher in local than cross-breed, while mortality and case fatality rate were higher in cross-breed, which is not statistically significant. Higher death was recorded in the local breed. The morbidity rate was higher in females (18.7%) than in males (9.68%) whose difference that was statistically significant (*P <* 0.05). The current findings concur with those of Ayelet et al. [25], Guyassa et al. [38], and Fayez and Ahmed [40]. Females have a higher morbidity rate because the lactation and pregnancy periods provoke physiological stress and reduced immunity. This physiological stress may result in temporary immunosuppression, increasing susceptibility to LSDV infection and clinical manifestation in female cattle.

Polymerase Chain Reaction (PCR) was the primary method for the rapid detection of LSDV. In our study, all 79 samples from 22 clinically affected cattle tested positive by real-time PCR (Darimu: 34/34; Bacho: 43/43; Yayo: 5/5), with negative controls showing no amplification. This 100% positivity is not a reflection of field prevalence but a direct consequence of the purposive sampling strategy, which deliberately selected animals meeting a strict clinical case definition and excluded asymptomatic or mildly affected animals. This sampling approach was designed to maximize diagnostic confirmation under outbreak conditions rather than estimate population-level prevalence.

Ct values ranged from 16.29 to 32.53, with low Ct values (16.5–25.5) in skin nodules indicating high viral DNA concentrations, supporting prior reports that skin biopsies contain higher viral loads [43]. Skin nodule biopsies yielded the lowest Ct value, confirming that this specimen contains the highest viral loads and is optimal for molecular confirmation. In contrast, whole blood and nasal swabs yielded significantly higher Ct values, indicating lower viral loads and a higher risk of false negatives in animals with low-grade viremia or later-stage infection. The variation in Ct values across sample types reflects differences in viral distribution and tissue tropism of LSDV. The significantly lower Ct values in skin nodules indicate active viral replication at the primary site of infection, while higher Ct values in blood and swab samples suggest lower systemic viral circulation during the clinical stage of disease. These findings align with OIE guidelines recommending skin lesions as the preferred sample type [30] and provide quantitative data to support field sampling protocols in Ethiopia.

Previous studies have reported lower detection rates using real-time PCR in clinically affected cattle, ranging from 83.87% to 88.8% in Egypt [44, 45]. Several factors may account for these differences, including variation in the timing of sample collection relative to the onset of clinical signs, the stringency of clinical case definitions, and the proportion of animals with active viremia at sampling. In the present study, sampling occurred early (within the first four weeks of clinical signs) and was restricted to animals meeting a strict clinical case definition, which likely contributed to uniformly positive results. However, the 100% positivity rate across all sample types, including blood, nasal, and ocular swabs, is at the upper extreme of reported findings and may be unusually high. This could reflect not only the targeted sampling approach but also the potential for overestimation due to selection bias. Consequently, this rate should not be interpreted as a typical field detection rate, and comparisons with studies that employed different sampling protocols or population-based designs should be made with caution. These findings demonstrate the diagnostic strength of lesion-based sampling, particularly skin nodules, for reliable molecular confirmation of LSD outbreaks under field conditions.

Seventeen real-time PCR-positive skin nodules with low Ct values were selected for virus isolation on lamb kidney (LK) cells. Of these, 23.5% (4/17; 1/10 from Darimu, 3/7 from Bacho) showed cytopathic effects (CPE). The four successful isolates originated from samples with the lowest Ct values, while 76.5% of samples failed to yield viable virus. This pattern indicates a clear relationship between viral load and successful virus recovery in cell culture, where lower Ct values are associated with higher likelihood of isolation success.

The relatively low virus isolation success rate compared to the high real-time PCR positivity (100%) reflects fundamental differences between molecular detection and virus culture techniques. Real-time PCR detects viral DNA regardless of infectivity, whereas virus isolation requires viable and replication-competent virus particles. Therefore, PCR positivity does not necessarily indicate the presence of an infectious virus. The failure to isolate the virus from a proportion of samples may be due to reduced viral viability resulting from sample handling, transport conditions, or the stage of infection at the time of sampling. Additionally, *Capripoxviruses* are known to be slow-growing and often require multiple passages before cytopathic effects become evident, which can reduce apparent isolation efficiency [30].

This finding has practical implications for studies requiring viable isolates (e.g., for genotyping, vaccine matching, or pathogenicity studies). Samples should be prioritized based on low Ct values, and collection should occur during the acute stage of infection when viral loads are highest. Additionally, failure rate may be due to the absence of live viruses in some samples and the inherent difficulty of *Capripoxvirus* isolation, which grows slowly and often requires additional passages [30].

The cytopathic effects observed, including cell rounding, aggregation of degenerated cells, and monolayer detachment, are consistent with previously described LSDV-induced changes in lamb kidney cell cultures. The delayed appearance of cytopathic effects during early passages, followed by more pronounced changes in later passages, further reflects the slow replication kinetics of LSDV in vitro. Similar CPE patterns have been reported in primary goat kidney cells [46] and bovine epithelial cultures [35]. The discrepancy between PCR detection and virus isolation highlights the superior sensitivity of molecular diagnostics for rapid outbreak confirmation, while virus isolation remains essential for confirming viral viability and enabling further molecular and antigenic characterization of circulating strains.

In the current study, the following limitations were acknowledged. First, the purposive sampling strategy, while appropriate for a confirmatory study, introduces selection bias that precludes generalization of the descriptive rates to the broader cattle population. This means that the calculated morbidity, mortality, and case fatality rates reflect only clinically suspected outbreak cases rather than the true population-level epidemiology. Second, the sample size (22 animals, 79 samples) was determined by outbreak case availability rather than statistical calculation, limiting the power of subgroup analyses. Third, the variable sample types collected across districts (e.g., no skin biopsies from Yayo) reflect real-world constraints but limit comparability. This may have introduced some variability in diagnostic yield across districts. Fourth, the study did not perform sequencing or genotyping of isolates, representing a missed opportunity to characterise circulating strains relative to vaccine strains. Fifth, no healthy control animals were included, so subclinical infection prevalence could not be estimated. Therefore, the study is primarily confirmatory and descriptive rather than epidemiological in a population-based sense.

In conclusion, LSD has a significant negative impact on livestock production through morbidity, mortality, and hide damage observed in the affected cattle population in Ilubabor Zone. Our study confirms the circulation of LSDV in Ilubabor Zone and demonstrates the feasibility of combining clinical, molecular, and virological methods for outbreak confirmation under field conditions in Ethiopia. For routine molecular diagnosis, skin nodules are strongly recommended based on their consistently lower Ct values and higher viral detection rate. The 100% PCR positivity among targeted samples validates the clinical case definition used in this study but underscores that such rates reflect the purposive sampling strategy rather than true field prevalence. LSDV was confirmed as the cause of the investigated outbreaks. The findings of this study are based on clinically suspected outbreak cases and therefore reflect confirmatory diagnostic outcomes rather than population-level epidemiology. To further improve understanding of LSD epidemiology in the region, additional studies are needed on strain distribution through genotyping and phylogenetic analysis of circulating LSDV strains. The results also highlight the importance of integrating consistent vaccination programs with laboratory-supported outbreak investigations and strengthening surveillance systems that consider the limitations of passive case reporting and non-random sampling approaches.

## Supplementary Information

Supplementary Material 1.

## Data Availability

All datasets generated or analyzed during this study are included in this published article.

## Abbreviations

AHI: Animal Health Institute
Ct: Cycle Threshold
EDTA: Ethylenediaminetetraacetic Acid
HRM: High-Resolution Melting
LK: Lamb Kidney
LSDV: Lumpy Skin Disease Virus
OIE: World Organization for Animal Health
PAs: Peasant Associations
PCR: Polymerase Chain Reaction
RT-PCR: Real-Time Polymerase Chain Reaction
VTM: Viral Transport Medium
