# Supplementary material for: Molecular detection and isolation of Lumpy Skin Disease Virus from outbreak cases in Ilubabor Zone, Oromia, Ethiopia
Source: BMC Microbiol. 2026 May 11;26:585. doi: 10.1186/s12866-026-05112-6 (PMC13330346; doi:10.1186/s12866-026-05112-6)
Supplement: Supplementary file 1 — Supplementary Material 1. [file 12866_2026_5112_MOESM1_ESM.doc]

A
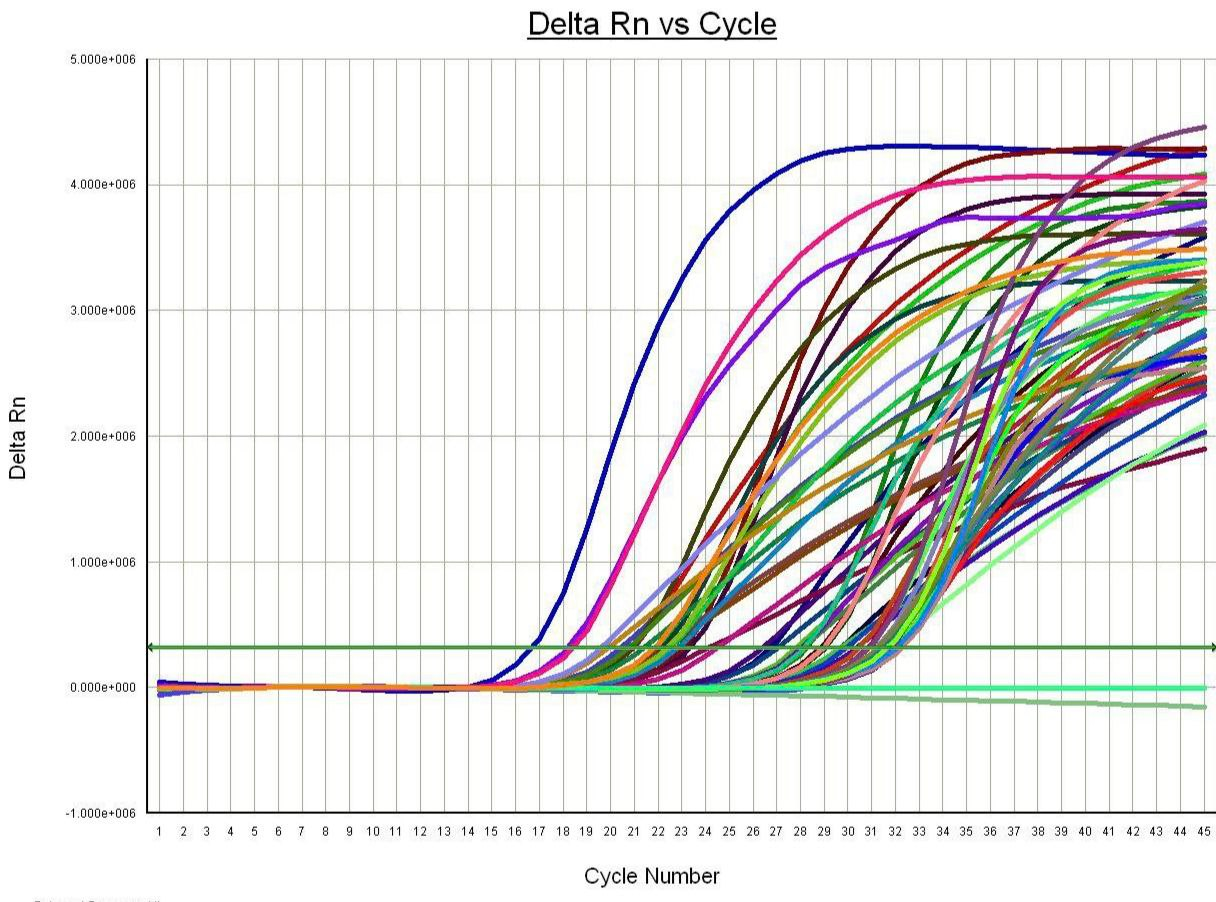


B
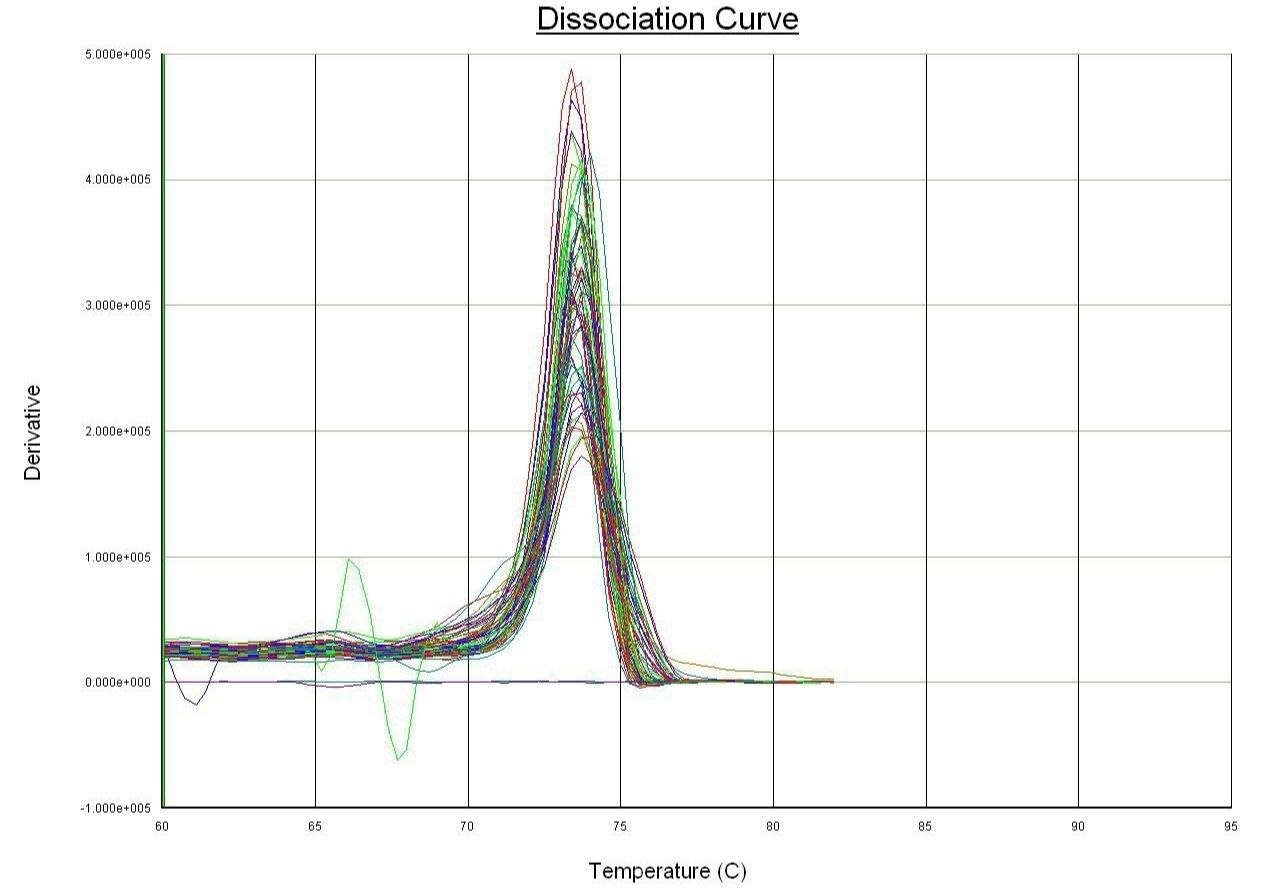


C
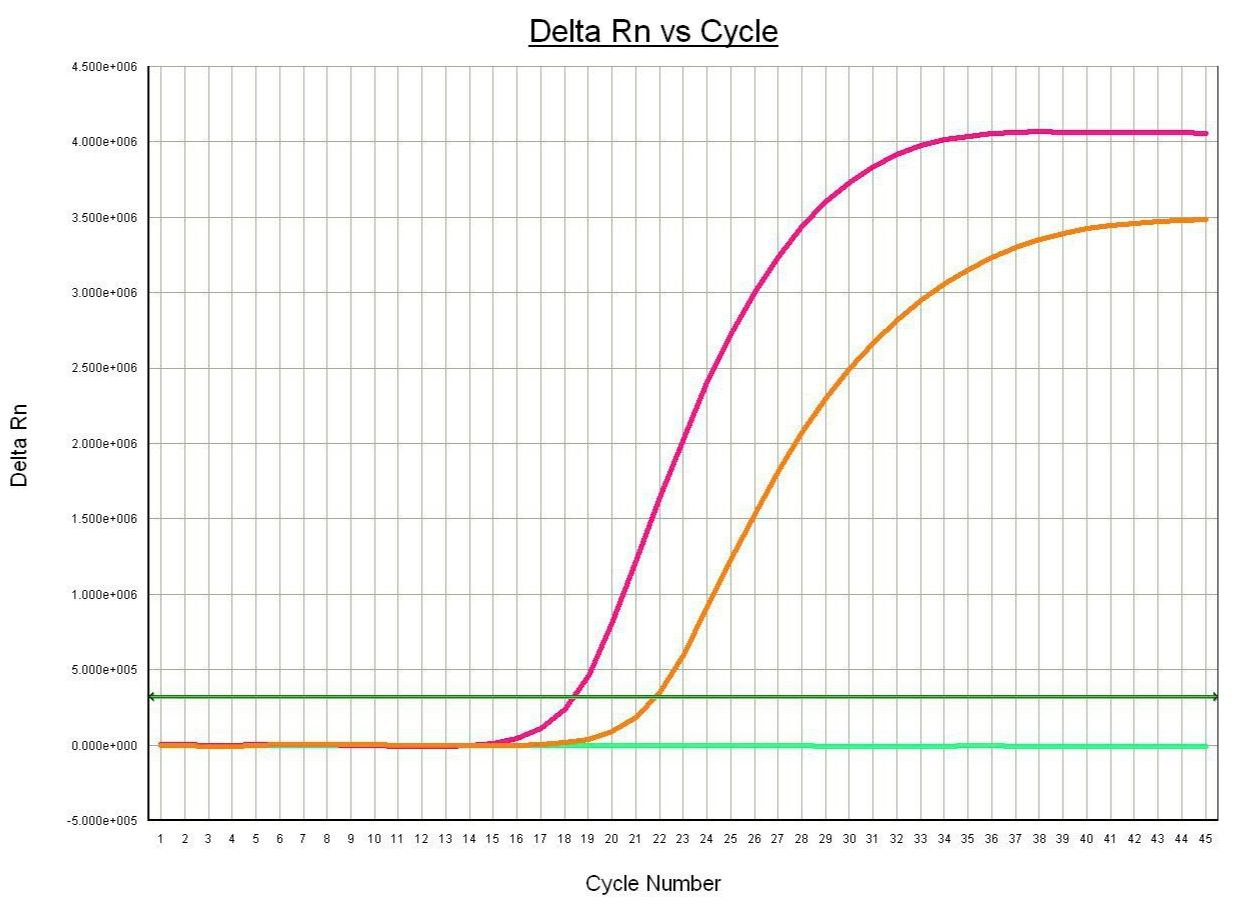


D
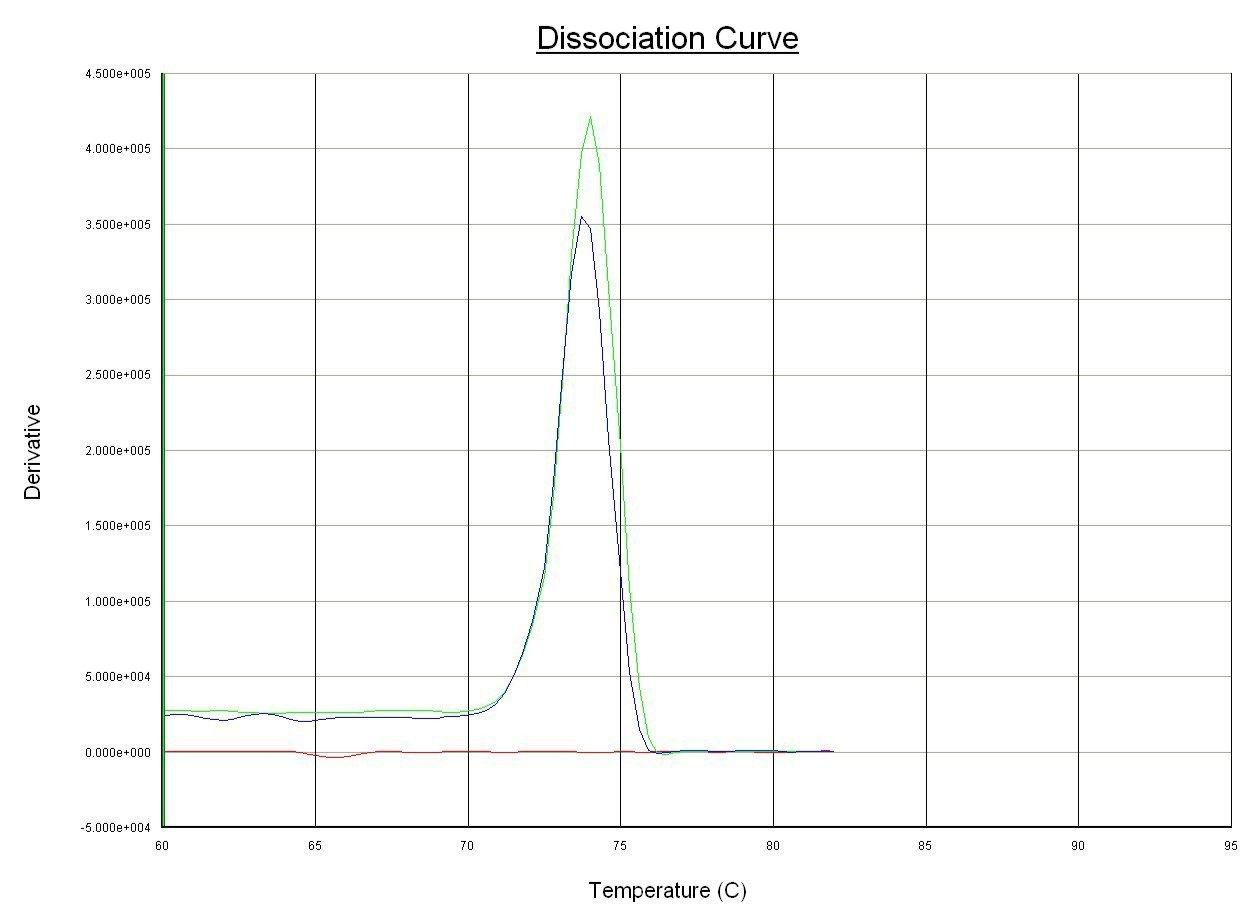


**Figure 3. **Real-time PCR results of LSDV-positive samples.**** (A, C) Amplification curves; (B, D) Melting (dissociation) curves.
